# Systemic glucocorticoid use and the occurrence of flares in psoriatic arthritis and psoriasis: a systematic review

**DOI:** 10.1093/rheumatology/keac129

**Published:** 2022-03-14

**Authors:** Nanette L A Vincken, Deepak M W Balak, André C Knulst, Paco M J Welsing, Jacob M van Laar

**Affiliations:** Department of Rheumatology & Clinical Immunology, University Medical Center Utrecht, Utrecht; Department of Dermatology, LangeLand Ziekenhuis, Zoetermeer; Department of Dermatology & Allergology, University Medical Centre Utrecht, Utrecht, The Netherlands; Department of Rheumatology & Clinical Immunology, University Medical Center Utrecht, Utrecht; Department of Rheumatology & Clinical Immunology, University Medical Center Utrecht, Utrecht

**Keywords:** PsA, glucocorticoids, psoriasis, symptom flare-up, therapeutics, systematic review

## Abstract

**Objectives:**

The use of systemic glucocorticoids (SGCs) is traditionally discouraged in the treatment of PsA and psoriasis due to the risk of psoriatic flares. However, despite this recommendation, SGCs are frequently prescribed for these patients. In this study we reappraise the old paradigm that SGCs are contra-indicated in the treatment of PsA and psoriasis.

**Methods:**

A systematic search of MEDLINE, EMBASE and the Cochrane Library databases was performed in November 2019 to identify articles on any SGC use compared with no use in the PsA and psoriasis population. Topical glucocorticoid treatment was excluded. Our two primary outcomes focused on the prescribing characteristics and the occurrence of any type of flare.

**Results:**

Our search yielded 4922 articles, and of these 21 full-text articles were eligible for inclusion. There were 11 retro- and prospective cohorts involving a total of 4,171,307 patients. Of these, 6727 (37.82%) of the patients with PsA and 1 460 793 (35.17%) of the patients with psoriasis were treated with any type of SGC. Ten observational/interventional studies did not report an increased risk or occurrence of psoriatic flares related to SGC use.

**Conclusion:**

Our results indicate that SGCs are frequently prescribed for PsA and psoriasis patients. The occurrence of psoriatic flares appears to be low upon SGC exposure. In patients with a clear indication for SGCs, e.g. in need of rapid anti-inflammatory therapy or bridging of therapies, the use of SGCs should be considered in view of the low risk of skin flaring. It remains of importance to weigh risks for short- and long-term SGC-related side effects in clinical decision making.

Rheumatology key messages

- Systemic glucocorticoids are frequently prescribed for the treatment of psoriatic arthritis and psoriasis.
- There is no solid evidence that systemic glucocorticoids increase the risk of psoriatic skin flaring.
- Systemic glucocorticoids should not be withheld for the treatment of PsA/psoriasis patients when indicated.

## Introduction

PsA is a heterogeneous, inflammatory autoimmune disease that is characterized by asymmetrical peripheral arthritis, dactylitis, enthesitis, SpA and psoriasis of the skin and nails [1, 2]. Approximately 20–30% of all psoriasis patients will eventually develop PsA [3]. Systemic treatment with DMARDs is essential in the management of PsA in order to prevent joint damage and erosions [4]. After initiation of a DMARD, it takes up to 3 months before treatment response can be observed in ∼40% of patients [5]. To bridge this initiation phase, systemic glucocorticoid (SGC) treatment can be given for rapid anti-inflammatory effects and to relieve pain [6]. This short-term complementary treatment has also been shown to improve long-term adherence and to improve drug survival in both psoriasis and PsA [7]. Furthermore, the addition of a SGC to current DMARD therapy gives better and faster clearance of psoriatic skin lesions and prolongs the drug-free remission period [8].

Despite these advantages, the use of SGCs for the treatment of PsA/psoriasis is traditionally discouraged by recent guidelines and textbooks due to the risks of psoriatic flares, yet they do not report evidence to support this recommendation [9–12]. Several guidelines refer to one and the same case series written in 1968, in which 19 of 104 patients developed generalized pustular psoriasis (GPP) after withdrawal of SGC therapy [13].

Several national health-care insurance databases have shown SGCs to be a frequently prescribed drug in the treatment of psoriasis in routine clinical practice. In Germany, the frequency of prescriptions for SGCs exceeds the amount prescribed for MTX, fumaric acid esters or biologics [14], and in the USA, SGC prescriptions are issued to psoriasis patients by 90% of dermatologists [15, 16]. This highlights the discrepancy between prescribing behaviour and current treatment guidelines.

Mrowietz and Domm [17] made an effort in 2012 to challenge the view of SGC use in psoriasis patients by highlighting the widespread use of these drugs without an observed increase in psoriatic flares. However, 10 years later, a shift in this old paradigm has not yet occurred. A systematic assessment of the evidence for the recommendation against the use of SGCs in psoriasis and PsA is lacking.

In this systematic review we aimed to: (1) address the general prevalence of SGC prescription in the PsA/psoriasis population, and (2) assess the risk and occurrence of psoriatic flares in PsA/psoriasis patients treated with SGCs.

## Methods

### Search strategy

A systematic patient/problem, intervention, comparison and outcome (PICO) search of MEDLINE, EMBASE and the Cochrane Library databases was performed in November 2019. The search strategy was constructed together with a medical librarian in order to identify any papers on SGC use compared with no use in the PsA/psoriasis population. Two primary outcomes of interest were: prevalence of SGC prescriptions and any type of psoriatic flare. We defined a flare as any type of reported exacerbation of the current psoriatic skin condition [e.g. using the psoriasis area and severity index (PASI), body surface area, clinical examination by a physician, or patients self-reporting that they had experienced a flare] or a morphological shift towards another phenotype (e.g. from psoriasis vulgaris towards erythrodermic psoriasis or psoriasis pustulosa). Search-terms used were PsA, Psoriasis, and Glucocorticoids combined with AND. Papers on topical treatment were excluded by using NOT as Boolean operator. A limit was set to English, Dutch and German language, and there was no time frame. All types of study design were considered, with the exception of case reports and case series. The PICO search strategy is presented in Supplementary Data S1, available at *Rheumatology* online.

### Study eligibility criteria

Eligibility outcome (1): studies describing the prevalence of SGC prescriptions must contain a population of unselected PsA/psoriasis patients and must report on the usage of SGCs within this population. Eligibility outcome (2): studies describing the occurrence of flares in patients 18 years and older with PsA/psoriasis starting, using or tapering SGCs. All doses and administration routes were considered, with the exception of topical treatment regimens. Studies in which patients concomitantly used conventional synthetic DMARDs (csDMARDs) or biologic DMARDs (bDMARDs) were included. Finally, for inclusion, the article was required to include a report on any type of psoriatic flare according to the definitions described above. All flares in response to start, dose maintenance, or tapering of SGCs were deemed to be of interest.

For all the articles obtained from the search strategy, the titles and abstracts were screened according to the inclusion criteria. If there was uncertainty about the article fulfilling the criteria, it was included for full-text screening, and eligibility was discussed with J.v.L./P.W. until consensus was reached. The references listed in all the included articles were screened to check for missing papers of interest.

### Data extraction and quality assessment

Three data extraction tables were created for each research question. Table 1 describes the general prevalence of SGC prescribing in the PsA/psoriasis population. Table 2 describes interventional studies reporting the risk of flares associated with SGC use in PsA/psoriasis patients compared with patients not using SGCs. Table 3 describes observational and interventional studies of PsA/psoriasis patients all using SGCs and the occurrence of flares in these populations. Publication types were clustered in order to give a clear distribution. Information on the aim of the study, number of patients, diagnosis, baseline demographics, SGC treatment regimen, and co-medication were extracted. Important outcomes were the number of patients who developed a psoriatic flare and a description of the flare according to the article.

**Table 1:** General prevalence of SGC prescriptions in the PsA/psoriasis population

Study		Study characteristics				Medication				
1st author (year)	Publication type	Diagnosis	*n*	Follow-up duration	Gender (female, %)	Type of steroid	Route of administration	Prescribing physician	Description of SGC prescriptions	Other
Al-Dabagh 2014	Retrospective cohort	Psoriasis	32 375	1989–2010	–	Prednisone, methylprednisolone, dexamethasone	PO, s.c.	93% by dermatologist; remainder by primary care physician	SGCs were prescribed at 650 000 visits; (3.1% of 21 020 000 psoriasis visits)	In 50% of cases, SGCs were prescribed alone; in 45% of the cases topical regimens were prescribed.
Armstrong 2017	Retrospective cohort	Psoriasis	1 700 000	2007–2012	–	Prednisone	PO	–	11.2% patients received prednisone as a first-line treatment	The number of patients treated with SGCs decreased in later lines of therapy.
Augustin 2011	Retrospective cohort	Psoriasis/PsA	26 338 psoriasis; 2319 PsA	2003–2007	42	Betamethasone, cloprednol, cortisone, deflazacort, dexamethasone, fluocortolone, hydrocortisone, meprednisone, methylprednisolone, prednisolone, prednisone and triamcinolone	PO	1191 prescriptions by general physician; 811 by internists; 259 by dermatologists	8.15% of psoriasis patients and 5.39% of PsA patients	Followed by MTX (*n* = 853), fumaric acid esters (*n* = 342); LEF (*n* = 168); retinoids (*n* = 110); CSA (*n* = 105). 44.18% of psoriasis and 2.5% of PsA patients received topical treatment
Dubreuil 2014	Retrospective cohort	Psoriasis/PsA	59 281 psoriasis; 4196 PsA	1986–2010	51 psoriasis; 50 PsA	Glucocorticoid use (NOS)	PO	General physician	4.3% of psoriasis patients and 8.2% of PsA patients	25% of psoriasis and 35.2% of PsA patients received topical treatment
Eun 2017	Retrospective cohort	Psoriasis	2 321 194	2010–2014	38	Methylprednisolone, prednisolone, dexamethasone, betamethasone, triamcinolone, other	PO, s.c.	General physician (93.9%), tertiary hospitals (2.2%), general hospitals (3.3%), small-sized hospitals (0.6%)	26.4% of psoriasis patients got a SGC prescription.	
Grassi 1997	Prospective cohort	PsA	180	1990–1992	58	Methylprednisolone, deflazacort, prednisone, betamethasone, dexamethasone, others	PO	–	24.4% of PsA patients were taking SGCs.	72.7% were simultaneously treated with a DMARD and 88.6% with a NSAID
Kavanaugh 2018	Prospective cohort	Psoriasis/PsA	7775 psoriasis; 1719 PsA; 4315 self-reported PsA	2007–2015	43 psoriasis; 49 PsA	Glucocorticoid use (NOS)	–	–	23.5% of all patients use or have used a SGC; Psoriasis 19.9%; PsA 33%; self-reported PsA 29.8%	48% of all patients use or have used an immunomodulator and 72.5% a biologic. 96.9% of all patients use or have used topical therapy
Lee 2016	Retrospective cohort	Psoriasis	6072	2001–2011	46	Glucocorticoid use (NOS)	–	–	20.27% of all patients currently use SGCs; 9.47% have used SGCs in the past.	7.32% of all patients have PsA; there are no subanalyses. 2.24% of patients are using MTX.
Madland 2005	Retrospective cohort	PsA	634	1999–2002	47	Prednisolone	PO, IA	Outpatient clinics	7.9% of PsA patients use oral SGCs; 40% of PsA patients had an IA injection.	40% currently use a DMARD and 1.8% use a biologic.
Rice 2018	Retrospective cohort	PsA	3932	2006–2015	–	Glucocorticoid use (NOS)	PO	Inpatient/outpatient clinics; general physician	All of the included PsA patients used SGCs.	26.9% used SGCs for >60 days; the remainder used SGCs intermittently.
Sinnathurai 2018	Prospective cohort	PsA	490	2003–2015	59	Prednisone, prednisolone	PO	–	25.7% of PsA patients use SGCs.	61% use MTX; 18.6% use LEF; 15.3% use SSZ; 4.1% use HCQ; 64.1% use a biologic.

SGC: systemic glucocorticoid; NOS: not otherwise specified; PO: oral; –: not specified.

**Table 2:** Papers directly comparing PsA and psoriasis patients using or not using SGCs in RCTs

Study			Baseline characteristics			Treatment and assessment of psoriatic flare or morphological shift description					
1st author (year)	Publication type	Aim of study	Dx	*n*	Age (Y, range)	SGC [dose, RoA]	Treatment duration	Co-medication	Reason for initiation of SGC	Skin flare or morphological shift during or after tapering of SGC	Percentage of flares
Carubbi 2016	RCT	Comparing efficacy and safety between SGC and TNF IA treatment	PsA	41	42.95 (31–68)	Triamcinolone (40 mg/month, IA), SGC (NOS)	3 months	Stable dose of anti-TNF in combination with one or more DMARDS	Refractory arthritis	No adverse events were reported during the 52-wk follow-up	0%
Gupta 2007	Open-label-RCT	Comparing efficacy and safety of MTX + betamethasone or MTX only	Psoriasis	40	39.63 (14–63)	betamethasone [3 mg/wk, PO]	Until complete clearance of lesions: 27.13 days (24.74–29.52)	15 mg MTX PO	Psoriasis	No flares after discontinuation (91.78 days in remission)	0%

Dx: diagnosis; Y: year; RoA: route of administration; RCT: randomized controlled trial; SGC: systemic glucocorticoid; NOS: not otherwise specified; PO; oral.

**Table 3:** Papers describing PsA and psoriasis patients all being treated with SGCs and the occurrence of flares in this population

Study			Baseline characteristics			Treatment and assessment of psoriatic flare or morphological shift description					
1st author (year)	Publication type	Aim of study	Dx	*n*	Age (Y, range)	SGC [dose, RoA]	Treatment duration	Co-medication	Reason for initiation of SGC	Skin flare or morphological shift during or after tapering of SGC	Occurrence of flares
Babino 2016	Retrospective cohort	Efficacy and safety of combination therapy with ETN	PsA/Psoriasis	37	59.43 (42–83)	Prednisone [25 mg/d, PO]	7 weeks (4–10 weeks)	ETN 25 mg 2/wk or 50 mg 1/wk, MTX	Cutaneous and/or articular inefficacy of ETN monotherapy	Safety profile was assessed: no skin flares	0%
Gregoire 2021	Retrospective cohort	Assess amount of any type of psoriasis flare associated with SGC use	Psoriasis	516	61.3 (SD 17.1)	Dexamethasone, fludrocortisone, hydrocortisone, methylprednisolone, prednisone [–, PO, injectable]	18.2 weeks (SD 64.2)	MTX (30), CSA(12), adalimumab (7), ETN (5), infliximab (3), ustekinumab (2)	–	16 flares: 15 mild plaque worsening, 1 erythrodermic	1.42% (95% CI, 0.72%, 2.44%)
Ganeva 2007	Prospective cohort	Assess adverse drug reactions of SGCd	Psoriasis	1041 (6 psoriasis)	48.9 (±18.9)	Methylprednisolone [–, PO], SGC NOS	Weeks – 4 years	NSAID; ACEi	Most frequently for autoimmune bullous dermatoses	None of the psoriatic exacerbation that led to hospitalization could be attributed to SGCs.	0%
Brody 1966	Single-arm trial	First study assessing efficacy and safety of triamcinolone treatment	Psoriasis	23	39.8	Triamcinolone [13 mg/2–3 weeks, i.m.]	Minimum 4 injections – maximum 50 injections over 3 years	Norethynodrel, chlordiazepoxide	Psoriasis	No flares or other adverse events were reported.	0%
Cohen 1959	Single-arm trial	Comparing efficacy and safety of different types of SGC IL and PO	Psoriasis	25	44.92 (21–71)	Triamcinolone [16 mg/d]; methylprednisolone [20 mg/d]; prednisolone [30 mg/d]; hydrocortisone [PO, IL]	4 months (1–7)	None	Psoriasis	No adverse events; up to 200 days in remission	0%
Haroon 2018	Single-arm trial	Comparing efficacy of i.m. triamcinolone on inflammatory back pain	Ax-PsA	40 (15 PsA/15 AS/10 control)	37.5	Triamcinolone [80 mg once, IA]	Once	60% of patients used DMARDs	Active PsA	No flares during follow-up period	0%
Coates 2016	RCT	Subanalyses of TICOPA trial assessing the occurrence of SGC-induced flares	PsA	206	45.5 (36–55)	Methylprednisolone 40 mg [5–120 mg, IA]; 120 mg [40–160 mg, i.m.]	Single/multiple administration	126 patients on DMARDs (90 MTX only, 30 combination)	Not specifically mentioned/inefficacy of current treatment	No adverse events reported, no significant change in PASI: patients did not report experiencing a flare. 10 patients had a PASI increase of ≥2	0%
Saviola 2007	Open-label RCT	Comparing efficacy and safety of deflazacort/methylprednisolone	PsA/RA	21 (7 PsA)	60 (33–73)	Methylprednisolone [4 mg/d]; deflazacort [7.5 mg/d] [PO]	1 year	MTX, CSA	Active PsA/RA	No flares during follow-up period	0%

Dx: diagnosis; Y: year; RoA: route of administration; SGC: systemic glucocorticoid; ETN: etanercept; PO; oral; NOS: not otherwise specified; ACEi: ACE-inhibitor; IL: intralesional; ax-PsA: axial psoriatic arthritis; RCT: randomized controlled trial; PASI: Psoriasis Area and Severity Index.

The methodological quality and risk of bias was assessed using the Agency for Healthcare Research and Quality (AHRQ) methodology checklist for cross-sectional and prevalence studies [18]. This manuscript was drafted using the Preferred Reporting Items for Systematic Reviews and Meta-Analyses (PRISMA) guidelines.

### Pooling of data

It was not possible to pool the quantitative data due to large heterogeneity in terms of the type of SGC, administration route, dosage, treatment duration, and psoriatic flare definition.

## Results

### Study characteristics

**Fig. 1:**
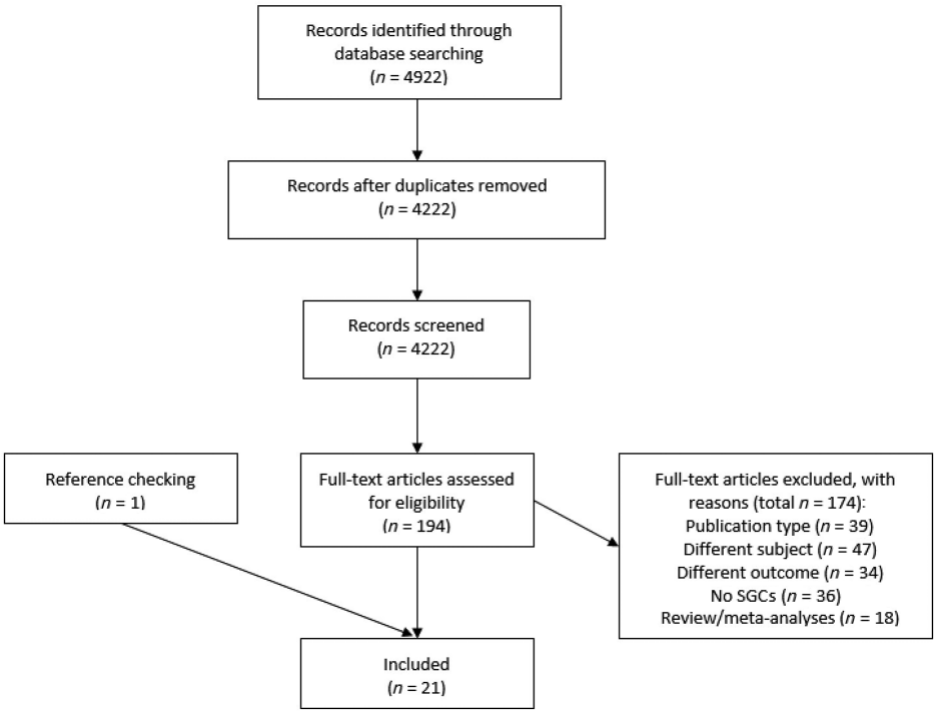
Prisma flow diagram of included articles SGCs: systemic glucocorticoids

The systematic literature search resulted in a total of 4222 unique articles after duplicate removal. Of these, 194 full-text articles were screened for eligibility, after which 21 articles were found to fulfil the selection criteria Fig. 1.

In general, male:female distribution was equal. The type of SGC, treatment duration, dosage, indication for prescription, and use of co-medication was heterogeneous for all included papers. Methylprednisolone (5–160 mg/d, with oral or IA/i.m./intralesional administration) was prescribed most frequently, followed by triamcinolone (13–80 mg/d, with oral or IA/i.m./intralesional administration). Less frequently, patients were treated with prednisone, prednisolone, dexamethasone, fludrocortisone, hydrocortisone, betamethasone and deflazacort. (Tables 2 and 3 show details on all SGCs used.) Reporting of other possible risk factors that could contribute or lead to a psoriatic flare was lacking.

### Assessment of SGC prescription prevalence for PsA and psoriasis patients

Eleven retro- and prospective cohort studies were included. Data were derived from National Health Care Insurance databases or online registries specifically designed to collect demographic data regarding PsA/psoriasis. The geographic origin of the data in the articles encompassed the USA, Germany, the UK, Korea, Australia, Norway, Italy and Taiwan. Sample sizes ranged between 180 and 2 321 194 patients, and the time period predominantly ranged from 2000 onwards. Two cohorts provided information from the period 1986–2010.

In summary, a total of 4 153 520 psoriasis and 17 787 PsA patients were analysed. A substantial proportion of patients have been treated with any type of SGC over the course of their disease: 1 460 793 psoriasis (35.17%) and 6727 PsA (37.83%) patients. Detailed study characteristics are shown in Table 1.

### Detailed descriptions of PsA/psoriasis populations treated with SGCs

Al-Dabagh et. al reported that SGCs were prescribed during 650 000 (3.1%) of the 21 020 000 total psoriasis visits, which was comparable with MTX, prescribed at 3.5% of all visits. When psoriasis was the sole diagnosis and no other comorbidities were present, 50% of all these prescriptions were SGC monotherapy. No other systemic treatment was added for the prevention of skin flares. Of the SGC prescriptions, 93% were prescribed by dermatologists [15]. In Germany, SGCs were the most frequently prescribed systemic drug in psoriasis patients (2774 of 34 728 patients, 7.98%), followed by MTX (853 of 34 728 patients, 2.46%). When correcting for potential comorbidities, such as PsA or other steroid-requiring comorbidities, 64% of all these prescriptions were made for the diagnosis of psoriasis only [14]. In psoriasis patients naïve for either systemic drugs or biologics, prednisone was prescribed for 75% of 254 000 patients, predominantly by primary care physicians as the first line of treatment. This frequency gradually decreased in later lines of therapy, when DMARD or biologic therapy become more prominent [16]. In a Korean study, 612 248 of 2 321 194 psoriasis patients (26.4%) were treated with SGCs in outpatient clinics. Patients who visited their primary care physician were more likely to be treated with SGCs then patients who visited tertiary hospitals [odds ratio (OR) 11.5, 95% CI (11.26, 11.72)] [19].

A similar high frequency of SGC prescriptions was seen among PsA patients. In an international longitudinal registry, 566 of 1719 PsA patients (33%) reported using SGCs at the time of enrolment [20]. Twenty-five percent of (126 of 490) PsA patients reported using SGCs in a voluntary Australian registry [21]. In a cohort of 3932 PsA patients, 26.9% received continuous treatment with SGCs, while 73.1% received intermittent treatment with SGCs [22]. In Norway, the proportion of patients treated with oral SGCs was lower (49 of 634 PsA patients, 7.9%), while the administration of IA steroid injections remained high (247 of 634 PsA patients, 40%) [2, 23]*.*

### The risk of a SGC-induced flare: two RCTs comparing patients using SGCs with those not using SGCs

Studies in which randomization occurs have a higher level of evidence. Only two RCTs were found directly comparing PsA/psoriasis patients using and not using SGCs. Both studies show no increased risk for psoriatic flaring associated with SGC exposure. One study assessed the safety and efficacy of IA injections with triamcinolone 40 mg or a TNFα inhibitor in 41 PsA patients with mono-arthritis. Patients received a triamcinolone injection once a month for 3 consecutive months and were followed for 52 weeks to assess joint flaring. All patients used concomitant DMARD or biologic therapy, and 63.4% of patients used oral SGCs at the time of intervention. No flares were reported in either group during or after SGC treatment [24]. In order to achieve faster clearance of psoriatic lesions and prolong the remission period, Gupta *et al.* conducted an open-label RCT including 40 patients, where one arm received weekly doses of 15 mg MTX and 3 mg betamethasone orally and the other MTX only until complete clearance of psoriatic lesions. After clearance of the lesions, treatment was ceased, and remission was monitored every 4 weeks until lesions started to reappear or new lesions formed. However, no flares were reported. Combination therapy was significantly better for both outcomes [8]. Table 2 shows all of the details of both RCTs.

### Observational and interventional studies describing flare occurrence in SGC-exposed PsA or psoriasis patients

Eight observational and interventional studies were used to explore the occurrence of SGC-related flares in PsA/psoriasis patients. Table 3 shows relevant details of the observational and interventional studies clustered by publication type. Two papers focused primarily on the research question: are patients exposed to SGC at greater risk of developing psoriatic flares. One recently published retrospective cohort included 516 patients using SGCs, with a median dose of 40 mg for a mean duration of 18.2 weeks. They identified a total of 16 psoriatic flares (1.42%) during or within 3 months of SGC exposure. Fifteen patients experienced mild worsening of plaque psoriasis, and one patient developed erythrodermic psoriasis. Six of these patients concomitantly took other medications known to induce psoriatic flares, (β-blockers, HCQ and quinacrine). The overall conclusion was that the frequency of flaring due to SGC exposure is low [25]. The other study involved a retrospective subanalysis of a RCT in which 206 PsA patients were allowed to receive IA/i.m. steroids as part of a tight control treatment regimen. A total of 161 episodes of SGC use in 101 patients were documented: 50 IA injections, with a median dose of 40 mg methylprednisolone and 111 i.m. injections with a median dose of 120 mg. A flare, defined as an increase in PASI score of ≥2, was seen in 10 patients. Overall, there was no significant PASI increase, and none of these patients self-reported experiencing an exacerbation of their skin symptoms during follow-up visits [26].

One retrospective cohort assessed the efficacy and safety of etanercept combination therapy: 4 out of 37 patients on etanercept were concomitantly treated with prednisone 25 mg/day for a mean duration of 7 weeks due to cutaneous inefficacy and/or articular inefficacy. PASI scores were monitored and no flares were reported [7]. In a prospective study aimed at identifying adverse drug reactions that led to hospitalization, none of the psoriasis vulgaris exacerbations could be attributed to SGC use [27]. In two single-arm trials, 23 and 25 psoriasis patients, respectively, received s.c. triamcinolone injections every 2–3 weeks until remission occurred or oral SGCs for an average of 4 months to determine the efficacy and safety. The longest treatment duration was 3 years. None of the participants experienced skin flaring during or after treatment [28, 29]. In an open-label controlled trial, 15 PsA patients with inflammatory axial involvement received a single dose of i.m. triamcinolone 80 mg to study improvement in inflammatory back pain. Sixty per cent of patients concomitantly used DMARDs, and no side effects were reported after a follow-up of 4 weeks [30]. In an open-label RCT study investigating the clinical efficacy and effects on bone metabolism of deflazacort or methylprednisolone, 7 PsA patients were enrolled. Patients were treated for 6 months with either deflazacort or methylprednisolone daily, after which cross-over took place to the other treatment arm. There were no flares reported during the follow-up period of the study [31].

The occurrence of psoriatic flares as reported over all studies ranged from 0 to 1.42%. This suggests that the risk of developing a flare after or during SGC exposure appears low.

### Critical review of Ryan and Baker’s case series on SGC-induced generalized pustular psoriasis

Guidelines discouraging SGC use in PsA/psoriasis mainly refer to a case series published by Ryan and Baker in 1968, in which 104 patients who presented with generalized pustular psoriasis (GPP) were assessed [13]. Cases were collected from records of the authors’ hospital (*n* = 24) and via questionnaires filled in by 43 dermatologists (*n* = 80), posing a high classification bias, because no standardized dermatological diagnostic criteria for GPP existed. Furthermore, the correctness of the completed questionnaires depended on each physician’s memory regarding his or her own case notes. In 19 of 104 patients who developed GPP, the suspected trigger was SGC use, as the exacerbation developed within a few days to weeks after tapering or withdrawal of the SGC. Another 10 patients were treated with SGCs because of already rapidly deteriorating psoriasis, which eventually progressed to GPP. These cases might have developed into pustular psoriasis spontaneously, independent of SGC use. Six other patients had not received SGCs before developing pustules, indicating that SGCs might not have been the cause of flares. Other possible causes for the development of GPP described were pregnancy, hypocalcaemia, infection, or topical use of potent CSs, or they may have been idiopathic [13]. To sum up, the paper by Ryan and Baker has a high risk of bias, and the recommendation originating from this paper is based on poor evidence and should not have been reiterated over the years without critically appraising the origin.

### Risk of bias

Two studies were of high quality, most were moderate, and four were of low quality. Typically, low-quality studies did not systematically report on their study protocol, nor did they elaborate on their attempt to control confounding. Moderate- to high-quality papers provided inclusion and exclusion criteria and the source of the data, elaborately reported potential confounding, and presented their primary outcomes clearly. One high-quality study applied blinding. A description of missing data was lacking in most articles. Assessment of the risk of bias of the studies is summarized in Table 4.

**Table 4:** Risk of bias

ARHQ Methodology Checklist	Al-Dabagh et al. 2014	Armstrong et al. 2017	Augustin et al. 2011	Babino et al. 2016	Brody, 1966	Carubbi et al. 2016	Coates, 2016	Cohen, 1959	Dubreuil et al. 2014	Eun et al. 2017	Ganeva et al. 2007	Grassi, 1998	Gregoire, 2021	Gupta and Gupta, 2007	Haroon et al. 2018	Kavanaugh et al. 2018	Lee et al. 2016	Madland et al. 2005	Rice et al. 2018	Saviola et al. 2007	Sinnathurai et al. 2018
1. Define source of information (survey, record review).	+	+	+	+	+	+	+	+	+	+	+	+	+	+	+	+	+	+	+	+	+
2. List inclusion and exclusion criteria for exposed and unexposed subjects (cases and controls) or refer to previous publications.	+	+	+	+	–	+	+	–	+	+	+	–	+	+	+	+	+	+	+	+	+
3. Indicate time period used for identifying patients.	+	–	+	+	–	–	+	–	+	+	+	+	+	–	–	+	+	+	+	–	+
4. Indicate whether or not subjects were consecutive, if not population based.	+	–	+	+	–	+	+	–	+	+	+	–	+	–	+	+	+	+	+	–	+
5. Indicate if evaluators of subjective components of study were masked to other aspects of the status of the participants.	NA	NA	NA	–	–	+	–	+	NA	NA	–	NA	–	–	–	NA	NA	NA	NA	–	NA
6. Describe any assessments undertaken for quality assurance purposes (e.g. test/retest of primary outcome measurements).	NA	NA	NA	NA	NA	NA	NA	NA	NA	NA	NA	NA	NA	NA	NA	NA	NA	NA	NA	NA	NA
7. Explain any patient exclusions from analysis.	+	–	+	+	–	+	–	–	+	+	+	–	+	+	–	–	+	–	+	+	–
8. Describe how confounding was assessed and/or controlled.	+	–	+	+	–	+	+	–	+	+	+	–	+	–	+	–	+	–	–	–	–
9. If applicable, explain how missing data were handled in the analysis.	NA	NA	NA	–	–	+	–	–	+	NA	–	–	–	–	+	–	–	–	NA	–	–
10. Summarize patient response rates and completeness of data collection.	+	+	+	+	+	+	+	–	+	+	+	+	+	+	+	+	+	+	+	+	+
11. Clarify what follow-up, if any, was expected and the percentage of patients for which incomplete data or follow-up was obtained.	NA	NA	NA	NA	–	+	+	–	+	NA	–	NA	–	–	–	NA	NA	NA	NA	+	NA
Total score	7	3	7	7	2	9	7	2	9	7	7	3	7	4	6	5	7	5	6	5	5

The quality of included articles was assessed using the Agency for Healthcare Research and Quality (AHRQ) methodology checklist for cross-sectional and prevalence studies. Article quality was assessed as follows: low quality = 0–3; moderate quality = 4–7; high quality = 8–11. Yes = +; No = –; Not applicable/Not specified = NA.

## Discussion

In this systematic review, we reappraised the old paradigm that the use of SGCs in PsA/psoriasis patients increases the occurrence of, or risk of developing, psoriatic flares. We found that SGCs are frequently used for the treatment of PsA/psoriasis in disregard of current treatment guidelines. Importantly, data describing the use of SGCs in this way mostly arise from relatively recent papers published between 2001 and 2015. By the extent of SGC usage, one would assume that the reported prevalence of SGC-related psoriatic flares would be much higher. Clinically, this is not the case. Evidence supporting advocation against the use of SGCs for psoriasis and PsA patients is mostly derived from case reports or case series [32–47]. In general, these publication types have a high risk of bias and provide low-quality evidence. Therefore, we feel that the original recommendation against the use of SGCs in this population is based on insufficient evidence.

After the publication of the case series by Ryan and Baker [13], the negative view of the use of SGCs in PsA/psoriasis patients was uncritically accepted. It is, however, important to mention that their paper has some important methodological limitations that influence the interpretation of the study results. The use of SGCs in psoriasis, and to some extent in PsA, is now traditionally discouraged and seen as malpractice. Several treatment guidelines for psoriasis and PsA directly refer to Ryan and Baker or to independent case reports/series as substantiation for the advice not to use SGCs [51–54]. Interestingly, the EULAR PsA treatment guidelines reiterate the recommendation that the use of SGCs might lead to psoriatic flaring, but mention that this recommendation is not substantiated by any evidence [12]. The GRAPPA PsA treatment guidelines indiscriminately highlight the risk of flaring without providing a direct source or critically reviewing this recommendation [9, 10]. It seems that, over the years, the recommendation to avoid SGCs for PsA/psoriasis patients has become generally accepted, and no effort has been made to critically reappraise this.

Only two papers report on flares associated with SGC use. In the paper of Coates *et al.* [26], in which a consensus definition of a psoriasis flare is lacking, a flare was defined as an increase in PASI of ≥2. Even though the PASI has reliable interobserver reproducibility [48, 49], it is not accurate for assessing mild psoriasis, and thus PASI might not be the best tool for monitoring flares [50]. Interestingly, all 10 patients with a PASI increase of ≥2 did not report experiencing a flare during follow-up visits. There seems to be a discordance between this clinical definition of a flare and the perception of the patients themselves. The other retrospective cohort specifically identified psoriasis patients exposed to SGCs and found that 1.42% experienced a mild worsening of their plaque psoriasis, while one patient developed erythrodermic psoriasis. An explanation for the low incidence of psoriatic flares could be that clinicians proactively take precautions to prevent flaring, for instance, by initiating combination therapy with topical or systemic DMARD therapy, and by tapering SGCs very gradually instead of by acute withdrawal.

SGCs are essential drugs that can rapidly reduce local or systemic inflammation in inflammatory diseases [55]. SGCs, whether given i.m., IA or orally, can be very beneficial in the early initiation phase of DMARD therapy in PsA or psoriasis to improve quality of life and reduce physical disability. Furthermore, SGCs have an anti-inflammatory effect by reducing pain, swelling and stiffness, and they induce immunosuppression that can ultimately prevent permanent joint damage [43, 56]. The combination of MTX and adjunctive SGCs has been shown to enhance faster psoriatic skin lesion clearance and to increase the drug-free remission period [8]. Drug survival is improved in etanercept-treated psoriasis and PsA patients who experience a loss of efficacy when temporarily co-treated with a SGC [7]. In addition to these benefits, it is generally known that SGCs also have the potential to cause adverse events, such as osteoporosis and -necrosis, infections, diabetes, cardiovascular disease, and suppression of the hypothalamic–pituitary–adrenal axis, especially when used for long-term treatment. However, when used thoughtfully, these adverse events are partially avoidable [57]. As SGCs pose multiple substantial beneficial effects, it would be undesirable to exclude them from the therapeutic armamentarium for PsA and psoriasis patients.

A limitation of this review is that the SGC prescription prevalence data is derived from insurance databases and that PsA/psoriasis patients were selected based on International Classification of Diseases codes. One cannot be certain that the SGCs prescribed at that time were solely meant for the treatment of PsA/psoriasis, or whether patients adhered to treatment. Even by filtering out patients with comorbid International Classification of Diseases codes that could explain SGC prescription (e.g. various rheumatologic conditions, urticaria, Crohn’s disease, COPD and asthma), there is no certainty for what indication the SGCs were really prescribed. Since the use of SGCs is traditionally discouraged for PsA/psoriasis, well-conducted RCTs are scarce, providing us with heterogeneous data in terms of SGC use, making it difficult to construct an evidence-based treatment recommendation. Finally, even though the search has been performed with a medical librarian, we cannot exclude the possibility that relevant articles have been missed.

This is the first systematic review questioning the old paradigm from a rheumatological and dermatological perspective. Prospective studies are needed to assess the real risk of flaring and to re-establish treatment guidelines discouraging SGC use. Considering how frequently SGC are being prescribed, the occurrence of psoriatic flares appears low and is only related to mild skin flaring, so we feel that SGC should not be withheld for the treatment of PsA/psoriasis patients when necessary. In patients with a clear indication for SGC use, e.g. those in need of rapid anti-inflammatory therapy or bridging of therapies, SGC should be considered in view of the low risk of skin flaring. It remains of importance to weigh the risks of short- and long-term SGC-related side effects in clinical decision making and possibly to treat patients in combination with a DMARD, biologic or topical treatment.

## Supplementary Material

keac129_Supplementary_Data

## Data Availability

The data underlying this article are available in the article and in its online supplementary material.
