# Supplementary material for: Systemic glucocorticoid use and the occurrence of flares in psoriatic arthritis and psoriasis: a systematic review
Source: Rheumatology (Oxford). 2022 Mar 14;61(11):4232–44. doi: 10.1093/rheumatology/keac129 (PMC9629346; doi:10.1093/rheumatology/keac129)
Supplement: keac129_Supplementary_Data [file keac129_supplementary_data.docx]

**SUPPLEMENTARY MATERIAL**

**Supplementary Data S1. PICO search strategy**

**Pubmed**

(("Psoriasis"[Mesh] OR psoriasis[Title/Abstract] OR psoriases[Title/Abstract] OR Arthritic Psoriasis[Title/Abstract] OR psoriatic Arthritis[Title/Abstract] OR Psoriasis Arthropathica[Title/Abstract] OR Psoriatic Arthropath*[Title/Abstract]) AND ("Prednisone"[Mesh] OR "Glucocorticoids"[Mesh] OR steroid*[Title/Abstract] OR "Glucocorticoids" [Pharmacological Action] OR prednisone[Title/Abstract] OR glucocorticoid*[Title/Abstract] OR glucocorticosteroid*[Title/Abstract] OR corticosteroid*[Title/Abstract])) NOT (topical[title/Abstract] OR "Administration, Topical"[Mesh])

**Embase**

('psoriasis'/exp OR 'psoriasis' OR 'psoriasis':ti,ab,kw OR 'arthritic psoriasis':ti,ab,kw OR 'psoriatic arthritis':ti,ab,kw OR 'psoriasis arthropathica':ti,ab,kw OR 'psoriatic arthropath*':ti,ab,kw) AND ('prednisone'/exp OR 'prednisone' OR 'glucocorticoids'/exp OR 'glucocorticoids' OR 'steroid*':ti,ab,kw OR 'prednisone':ti,ab,kw OR 'glucocorticoid*':ti,ab,kw OR 'glucocorticosteroid*':ti,ab,kw OR 'corticosteroid*':ti,ab,kw) NOT ('topical':ti,ab,kw OR 'administration, topical'/exp OR 'administration, topical')

#1 AND [embase]/lim AND [medline]/lim

#1 AND [embase]/lim AND [medline]/lim AND 'Article'/it
